# Functional Response of *Harmonia axyridis* to the Larvae of *Spodoptera litura*: The Combined Effect of Temperatures and Prey Instars

**DOI:** 10.3389/fpls.2022.849574

**Published:** 2022-07-01

**Authors:** Yasir Islam, Farhan Mahmood Shah, Ali Güncan, John Paul DeLong, Xingmiao Zhou

**Affiliations:** ^1^Hubei Insect Resources Utilization and Sustainable Pest Management Key Laboratory, College of Plant Science and Technology, Huazhong Agricultural University, Wuhan, China; ^2^Department of Entomology, Faculty of Agricultural Sciences and Technology, Bahauddin Zakariya University, Multan, Pakistan; ^3^Department of Plant Protection, Faculty of Agriculture, Ordu University, Ordu, Turkey; ^4^School of Biological Sciences, University of Nebraska–Lincoln, Lincoln, NE, United States

**Keywords:** biocontrol, space clearance rate, handling time, lepidoptera, coccinellids, warming, predation

## Abstract

Functional responses are central to predator–prey dynamics and describe how predation varies with prey abundance. Functional responses often are measured without regard to prey size (i.e., body mass) or the temperature dependence of feeding rates. However, variation in prey size within populations is ubiquitous, and predation rates are often both size and temperature-dependent. Here, we assessed functional responses of larvae and adult *Harmonia axyridis* on the 1st, 2nd, and 3rd instars of the prey *Spodoptera litura* across a range of temperatures (i.e., 15, 20, 25, 30, and 35°C). The type and parameters of the functional responses were determined using logistic regression and fitted to the Roger's random predator equation. The magnitude of predation varied with the predator and prey stage, but prey predation increased with warming and predator age. Predation by the female and 4th instar of *H. axyridis* on the 1st instar of prey was greater, followed by the 2nd and 3rd instar of prey *S. litura*. No predation occurred on the larger prey for the 1st, 2nd, and 3rd instars of *H. axyridis*. The larvae and adult *H. axyridis* produced a type II (hyperbolic) functional response curve across all temperatures and the three prey types they consumed. Space clearance rates, handling time, and maximum predation rates of *H. axyridis* changed with temperature and prey size, increasing with temperature and decreasing with prey size, suggesting more predation will occur on younger prey. This study indicates an interactive role of temperature and prey/predator size in shaping functional responses, which might complicate the planning of effective biocontrol strategies against this serious pest.

## Introduction

Trophic interactions are central to community ecology and ecosystem stability (Wang and Zou, 2017) by structuring food webs (Mcintosh et al., 2017), driving population dynamics (Noman et al., 2021), and shaping landscapes of fear (Fardell, 2022). These ecological functions, however, are regulated by species interaction strengths within food webs (Novak and Wootton, 2010). Many biotic and abiotic factors (King et al., 2021), including temperature, arena size, species identity, body mass, and offensive or defensive structures (DeLong, 2014; Li et al., 2018; Uiterwaal and DeLong, 2020), influence species interaction strengths. Measuring functional responses, i.e., the relationship between foraging rate and prey abundance, can thus help improve our understanding of predator–prey relationships and use them to generate biocontrol programs that are effective and sustainable (Holling, 1959; DeLong and Uiterwaal, 2021).

A fundamental factor influencing the biological rates of most ectotherms is the temperature (Kim et al., 2020). The impact of temperature on biological rates extends from biochemical reactions to individual physiology to nutrient cycling in ecosystems (Brown et al., 2004; Rall et al., 2012; Yvon-Durocher et al., 2012; Amarasekare, 2015; Islam et al., 2022). The temperature has the potential to modify species interaction strengths and alter energy distribution across food webs. With climate change becoming a major challenge worldwide, interest in determining the effect of temperature on ecological systems has grown (García et al., 2018). Insects are ectotherms, and hence their growth, maturation, reproduction, and trophic interactions can be modified by their thermal surroundings (Amarasekare, 2015; Govindan and Hutchison, 2020). These effects indicate that pest management strategies relying on insects as biocontrol agents against arthropod pests can be altered by temperature changes.

There are three typical types of functional responses (Holling, 1959; DeLong, 2021): a linear rate of predation up to a constant plateau (type I), hyperbolic (type II), or sigmoid (type III), based upon whether the functional response parameters, i.e., the coefficients of space clearance rate (*a*) and prey handling time (*T*_*h*_), vary with prey abundance. The space clearance rate corresponds to the area cleared of prey per predator per unit of time, whereas handling time measures the loss in search time associated with killing, consuming, and digesting the prey (Pervez, 2005). A high space clearance rate and low handling time are general characteristics of a successful biocontrol agent (DeLong and Uiterwaal, 2021).

Functional responses often are estimated without regard to body size or other traits (Jalali et al., 2010; DeLong et al., 2021). However, variation in prey size owing to ontogeny and individual variation is ubiquitous, and predation rates typically depend on the size of the predator, the prey, or both (Cogni et al., 2002; Mccoy et al., 2011). This variation stems from the effect of predator and prey body size on the functional response parameters (i.e., handling time and space clearance rate) (DeLong, 2014; Uiterwaal and DeLong, 2020; Buba et al., 2021). Generally, handling time increases as prey size increases and decreases as predator body size increases. Space clearance rate may increase with predator body size, while the effect of prey body size on space clearance rate is more variable (Uiterwaal and DeLong, 2020; Buba et al., 2021). Similarly, functional responses often are estimated at only one temperature, despite the widespread effect of temperature on functional response parameters. Across many systems and in several focused studies, the temperature has a unimodal effect on both space clearance rate and handling time (Uszko et al., 2017; Uiterwaal and DeLong, 2020). In smaller comparative studies, however, the temperature may have a monotonic effect on functional responses (Buba et al., 2021; DeLong and Uiterwaal, 2021). It is imperative to consider the temperature in biological control strategies, especially for biological control with non-indigenous natural enemies (Islam et al., 2020, 2021).

*Spodoptera litura* F. (Lepidoptera: Noctuidae) is a noxious polyphagous pest with a worldwide distribution, attacking many agricultural, horticultural, and ornamental plants (Atwal and Dhaliwal, 2009). It feeds on an extensive host range of 389 species in 109 plant families (Qin et al., 2006). The pest lays eggs in batches, yielding a large population of hatchlings that are gregarious and spread over nearby plants as they grow. The hatchlings develop through 5-7 larval instars (Rao et al., 1989), during which they can inflict huge losses on sensitive crops (Razaq et al., 2019; Shah et al., 2020). The potential for whole crop failures causes farmers to heavily apply pesticides to their crops (Saleem et al., 2016; Shah et al., 2017, 2019). As a result, the pest has developed resistance to synthetic pesticides, leading to control failures and outbreaks (Xie et al., 2010; Xu et al., 2012). With over 638 resistance reports against 39 active pesticide ingredients (Whalon et al., 2012), the management of this pest remains a challenge.

It is therefore necessary to consider alternative approaches, such as biological control with predators, to suppress this pest. About 131 species of natural enemies are reported to feed on *S. litura* (Rao et al., 1993). *Harmonia axyridis* (Pallas) (Coleoptera: Coccinellidae) is an important predator of several economically important hemipterans (Huang et al., 2019; Gao et al., 2020) and lepidopterans (Koch et al., 2003; Uiterwaal and DeLong, 2018, 2020; Islam et al., 2020). Recently, in companion studies, we showed temperature-dependent predation and functional responses of *H. axyridis* to eggs of *S. litura*, but there are no prior estimates of the functional response of *H. axyridis* to the larval stages of *S. litura*. This study estimated the functional responses of *H. axyridis* foraging on five larval stages of *S. litura* across a range of temperatures and the body size of the predators. We tested the hypothesis that the functional response interactively depends upon prey size, predator growth stage, and temperature.

## Materials and Methods

### Rearing of *H. axyridis*

In January 2019, colonies of *H. axyridis* and *Acyrthosiphon pisum* Harris (Hemiptera:Aphididae) were obtained from a collection of adults from a stock culture at the Key Laboratory of Hubei, Insect Resources Utilization and Sustainable Pest Management, located at Huazhong Agricultural University (HZAU), Wuhan, China. The predator was fed with *A. pisum*, and both cultures were maintained on *Vicia faba* (L.) Fabaceae (Leguminosae) bean plants inside a growth chamber at 26 ± 1°C, 60–70% RH, and 16:8 h (light:dark) photoperiod (Knapp and Nedvěd, 2013).

Thirty pairs of *H. axyridis* adults were mated inside 9 ×5 cm^2^ plastic cups (Yongxin and Plastic Products Guangzhou Co., Ltd: Model No 277), which had fine mesh covering the top for ventilation and crumpled filter papers at the bottom as an oviposition substrate. The adults of *H. axyridis* were kept inside plastic cups and fed with *A. pisum*. The oviposition was checked daily. Eggs were removed daily and kept in 6 cm Petri dishes with moistened cotton. Post-emergence hatchlings were subsequently maintained inside similar plastic cups containing aphids for prey that were refreshed daily. Upon reaching sexual maturity, the adults were sexed and the developed progeny was used to establish population culture. In this way, the whole population culture was maintained throughout the trial with individuals from each stage required for this study.

### Rearing of *Spodoptera litura*

The *S. litura* egg masses were purchased from Henan Jiyuan Baiyun Industrial Co., Ltd, China, in February 2019, and incubated inside the growth chamber at 26°C and 70 ± 5 % RH under a 12:12 h (light: dark) photoperiod. Post-emergence hatchlings were fed with a standard artificial diet (Ul Haq et al., 2015) and cultured until they reached the final instar stage inside mesh-covered transparent circular glass jars (1 L). The artificial diet (semi-solid) consisted of ascorbic acid (2.35 g), distilled water (550 ml), kidney bean flour (150 g), agar (8.4 g), sorbic acid (0.75 g), yeast powder (24 g), methyl-4-hydroxy benzoate (1.5 g), formaldehyde solution (1 ml), and streptomycin (0.75 g). The final instars were isolated and subsequently cultured through maturity inside similar jars that contained moist cotton balls as a pupation media and a 15% glucose solution for food. A clean piece of paper completely covered the jar's bottom as an oviposition substratum (Chang et al., 2020). Oviposition was checked daily, and post-emergence hatchlings were reared together to obtain uniform sets of individuals of the same age. The population culture was established separately for the 1st, 2nd, and 3rd instars. The length of the 1st instar of prey varied from 1.1 to 1.7 mm, whereas that of the 2nd and 3rd instars varied from 2.4 to 3.9 mm and 7.6 to 8.5 mm, respectively (Ramaiah and Maheswari, 2018). The mean weights were 7.31 ×10^5^ ± 2.99 ×10^−5^ g/larva for 1st instar of prey, 2.95 ×10^−4^ ± 1.20 ×10^−4^ g/larva for 2nd instar of prey, and 1.37 ×10^−3^ ± 5.58 ×10^−4^ g/larva for 3rd instar of prey *S. litura*.

### Functional Response Experiment

The predators were fed with aphids during development. It was therefore necessary to remove maternal prey effects and acclimatize the predator to the new prey. This was approached by rearing *H. axyridis* on 1st instar of *S. litura* for at least two complete generations. The 1st, 2nd, 3rd, and 4th instars, and adult (male and female) individuals of *H. axyridis* used in this study were of the approximate same age (0-6 h) and were starved singly to stimulate hunger. The starvation period was 6 h for the 1st instar and 24 h for the subsequent instars/stages (Islam et al., 2020). During starvation, humidity was provided inside Petri dishes through moist cotton. The minimum time required between molts was always greater than 48 h for the tested temperatures. This suggests little chance for molting to interfere with functional response experiments over a 24-h period.

Experiments were conducted at five constant temperatures (i.e., 15, 20, 25, 30, and 35°C) under fixed settings of 65 ± 5 % RH and photoperiod of 16:8 (light: dark) inside a computer-controlled growth chamber (Shanghai Xinmiao Medical Device Manufacturing Co., Ltd: Model No QHX-250 BSH-III). The temperature regimes reflect thermal conditions experienced by *H. axyridis* in various protected plantations and field crops in tropical and temperate areas (Brown et al., 2011). Tomato, *Solanum lycopersicum* L. (Solanales: Solanaceae), is a suitable host plant for *S. litura* (Bano and Muqarab, 2017). The experiments were conducted on circular tomato leaflets, cut to fit inside a 9-cm diameter dish, and wrapped at petioles with moist cotton to prevent drying. Three independent prey/predator systems were established in this study. In the first system, predators in all growth stages were allowed to feed on the 1st instar of *S. litura*. The densities of 1st instar of prey offered to the 1st and 2nd instars of *H. axyridis* were 3, 6, 10, 15, 20, 25, 35, and 50 larvae/arena, and 5, 10, 20, 30, 40, 50, 60, and 70 larvae/arena, respectively, whereas 3rd and 4th instars, and adult male and female were offered the densities of 25, 50, 75, 100, 150, 200, 250, and 300 larvae/arena. In the second system, older predator instars/stages (i.e., 3rd and 4th instars, and both adults) were given only 2nd instar of *S. litura* at the densities of 5, 10, 20, 30, 50, 80, 100, and 120 prey larvae/arena. Here, 1st and 2nd instars of *H. axyridis* were excluded from the candidate list, as they were not found to be able to feed on 2nd instar of prey in the preliminary assessments. In the third system, the predators (i.e., 4th instar and both adults) were offered with the 3rd instar of *S. litura* at the densities of 2, 4, 8, 16, 32, 40, 45, and 50 prey larvae/arena. Here, 1st, 2nd, and 3rd instars of *H. axyridis* were excluded from the candidate list, as they could not consume the 3rd instar of prey. A control treatment that only contained prey but no predator was also established for each prey/predator system to account for natural mortality. Within each of these systems, the selection of prey densities offered to the predator was based on preliminary assessments. All experimental treatments were replicated 10 times, and the control treatment had five replicates. One predator was placed on the tomato leaflet inside the arena (9-cm diameter) using a fine camel hairbrush. Prey was pre-released about 30 min before the predator to settle over the substrate. The escape of predator and prey was prevented by wrapping parafilm around the edges of the arena. Larvae consumed by *H. axyridis* were not replaced during the entire experiment. After 24 h, the larval and adult *H. axyridis* were removed from the arenas, and the number of prey caterpillars, either damaged, killed, regurgitated, or consumed, was recorded for predation assessment.

### Data Analyses

The control mortality of all stages of prey *S. litura* (1st, 2nd, and 3rd instars) was none to very low (<2%) across all five temperatures, and therefore data were not adjusted for control mortality. Predation of prey by *H. axyridis* was analyzed using generalized linear models (*GLM*) with a binomial distribution and a logit link function. Temperature, growth stage, prey size, density, and their interactions were considered as fixed effects. The dependent variable was *N*_e_. As three prey sizes were used, and as prey densities could be confounded by prey size (Uszko et al., 2020), we used the mean weight of each prey in each replicate as a covariate. Non-normal distributions of the data (*P* > 0.05) were tested by Kolmogorov–Smirnov test. Factors and interaction effects were assessed using the likelihood ratio (LR) chi-square test with a 95 % percent confidence interval (CI). Following the detection of significant effects of prey size (main/interaction), we further tested the effects of temperature, growth stage, density, and their two-way or three-way interactions within each of the three prey sizes offered. Because several tests were carried out to determine the effects of temperature, predator stage, prey instar, and their interaction, we employed the Bonferroni correction to correct for significance, i.e., dividing the standard significance criterion (*P* <0.05) by the number of tests evaluated. Statistical analyses were performed by using R software (v4.1.2) (R Core Team et al., 2022) for *GLM* analysis and “car” (Fox and Weisberg, 2011) packages for the likelihood ratio (LR) chi-square test.

#### Functional Response Assays

We used the two-step procedure recommended by Juliano (2001) to determine the form and parameters of a functional response. The form of the functional response curve was first determined by fitting polynomial logistic regression on the proportion of larvae consumed across larvae densities, as described by:


(1) $$\begin{array}{l} \frac{N_{e}}{N_{0}}=\frac{\exp\left(P_{o}+\;P_{1}N_{0}{+\;P}_{2}N_{0}^{2}{+\;P}_{3}N_{0}^{3}\right)}{1+\exp\left(P_{o}+\;P_{1}N_{0}{+\;P}_{2}N_{0}^{2}{+\;P}_{3}N_{0}^{3}\right)} \end{array}$$

where *N*_e_ and *N*_0_ represent the number of prey killed or consumed and initial prey density, respectively, and $\frac{N_{e}}{N_{0}}$ represents the proportion of prey that was consumed. *P*_0_, *P*_1_, *P*_2_, and *P*_3_ are the constant, linear, quadratic, and cubic coefficients of the regression, respectively. We started with a cubic model for fitting the polynomial logistic regression; however, since the parameters of this model are non-significant (*P* > 0.05), we then eliminated the cubic term $N_{0}^{3}$ and simplified polynomial logistic regression to fit with the quadratic coefficient. These coefficients were calculated using generalized linear models (*GLM*) with a binomial distribution and a logit link, and a maximum likelihood function with R software (v4.1.2) (R Core Team et al., 2022). Type II and type III functional responses can be distinguished based on the shape of the curves obtained by fitting Equation 1 to the proportional predation data. A type II response is described by a negative linear estimate (decreasing proportional predation with increasing prey density), and a type III functional response by a positive linear estimate followed by a negative quadratic term (increasing but then decreasing proportional predation with increasing prey density) (Juliano, 2001). The type II response indicates that predation declines monotonically with prey density, and a type III response shows that the proportional prey predation is positively density-dependent (Holling, 1959). The logistic regression indicated that the data fit a type II functional response (*P*_1_ <0) at all stages and tested temperatures (see Table 1). Thereafter, Roger's “random predator equation” (Equation 2) (Rogers, 1972) was applied, which is appropriate for our experiment without prey replacement (Juliano, 2001). This model is as follows:


(2) $$\begin{array}{l} N_{e}=N_{0}\;\left[1-\exp\left(a\left(T_{h}N_{e\;}-T\right)\right)\right] \end{array}$$

where *N*_*e*_ and *N*_0_ again represent the number of prey consumed and offered, respectively, α is the space clearance rate, *T*_*h*_ is the handling time, and *T* is the total time available for predators to forage (i.e., 24 h for this study), and it is solved using Lambert's transcendental equation (Bolker, 2008). We used the “frair” R software package to determine the coefficients of space clearance rate and handling time (Pritchard et al., 2017). The 95% confidence intervals (CIs) for space clearance rate and handling time were generated by nonparametric bootstrapping (*n* = 10,000) again by using “frair” R package. Then we compared fitted coefficients in which non-overlapping 95% CIs are considered significantly different (Pritchard et al., 2017).

**Table 1:** Maximum likelihood estimates from logistic regression analyses of the proportion of prey eaten by different stages of *Harmonia axyridis* preying upon 1st, 2nd, and 3rd instars of prey *Spodoptera litura* at various temperatures.

**Predator** **stage**	**Temperature** **(°C)**	**Prey** **stage**	**Intercept**				**Sig^**a**^**	**Linear**				**Sig^**a**^**	**Quadratic**				**Sig^**a**^**
			**Estimates**	**S.E**.	**Z-value**	***Pr(>|z|)***		**Estimates**	**S.E**.	**Z-value**	***Pr(>|z|)***		**Estimates**	**S.E**.	**Z-Value**	***Pr(>|z|)***	
1st larvae	15	1st	0.760	0.237	3.207	1.341 ×10^−3^	**	−0.107	0.019	−5.740	9.49 ×10^−09^	***	1.1233 ×10^−3^	3.069 ×10^−4^	3.660	2.52 ×10^−4^	***
	20	1st	1.470	0.244	6.024	1.7 ×10^−9^	***	−0.133	0.019	−7.161	8.03 ×10^−13^	***	1.4818 ×10^−3^	3.006 ×10^−4^	4.929	8.28 ×10^−7^	***
	25	1st	2.573	0.288	8.946	<2 ×10^−16^	***	−0.135	0.020	−6.752	1.46 ×10^−11^	***	1.3643 ×10^−3^	3.078 ×10^−4^	4.432	9.33 ×10^−6^	***
	30	1st	3.387	0.371	9.134	<2 ×10^−16^	***	−0.121	0.024	−4.986	6.16 ×10^−7^	***	1.027 ×10^−3^	3.57 ×10^−4^	2.877	4.01 ×10^−3^	**
	35	1st	7.336	0.883	8.312	<2 ×10^−16^	***	−0.267	0.050	−5.329	9.9 ×10^−8^	***	2.5379 ×10^−3^	6.678 ×10^−4^	3.800	1.45 ×10^−4^	***
2nd larvae	15	1st	0.944	0.216	4.371	1.24 ×10^−5^	***	−0.081	0.011	−7.139	9.42 ×10^−13^	***	6.132 ×10^−4^	1.324 ×10^−4^	4.633	3.61 ×10^−6^	***
	20	1st	2.093	0.239	8.765	<2 ×10^−16^	***	−0.095	0.012	−8.232	<2 ×10^−16^	***	7.121 ×10^−4^	1.287 ×10^−4^	5.532	3.17 ×10^−8^	***
	25	1st	3.632	0.337	10.785	<2 ×10^−16^	***	−0.107	0.015	−7.299	2.91 ×10^−13^	***	7.332 ×10^−4^	1.521 ×10^−4^	4.821	1.43 ×10^−6^	***
	30	1st	4.880	0.467	10.459	<2 ×10^−16^	***	−0.125	0.019	−6.524	6.84 ×10^−11^	***	7.868 ×10^−4^	1.897 ×10^−4^	4.147	3.37 ×10^−5^	***
	35	1st	9.261	0.903	10.252	<2 ×10^−16^	***	−0.265	0.034	−7.784	7.01 ×10^−15^	***	1.9271 ×10^−3^	3.111 ×10^−4^	6.194	5.88 ×10^−10^	***
3rd larvae	15	1st	0.224	0.106	2.113	3.46 ×10^−02^	*	−0.016	0.001	−11.363	<2 ×10^−16^	***	2.71 ×10^−5^	4.021 ×10^−6^	6.740	1.58 ×10^−11^	***
	20	1st	0.931	0.103	9.017	<2 ×10^−16^	***	−0.022	0.001	−15.753	<2 ×10^−16^	***	3.985 ×10^−5^	3.852 ×10^−6^	10.345	<2 ×10^−16^	***
	25	1st	1.207	0.102	11.890	<2 ×10^−16^	***	−0.021	0.001	−16.070	<2 ×10^−16^	***	3.795 ×10^−5^	3.621 ×10^−6^	10.480	<2 ×10^−16^	***
	30	1st	2.184	0.110	19.856	<2 ×10^−16^	***	−0.019	0.001	−15.090	<2 ×10^−16^	***	3.202 ×10^−5^	3.341 ×10^−6^	9.583	<2 ×10^−16^	***
	35	1st	2.979	0.129	23.060	<2 ×10^−16^	***	−0.021	0.001	−15.040	<2 ×10^−16^	***	3.409 ×10^−5^	3.588 ×10^−6^	9.500	<2 ×10^−16^	***
	15	2nd	−0.760	0.177	−4.297	1.73 ×10^−5^	***	−0.032	0.006	−4.982	6.29 ×10^−7^	***	1.42 ×10^−4^	4.59 ×10^−5^	3.094	1.97 ×10^−3^	**
	20	2nd	0.777	0.237	3.280	1.04 ×10^−3^	***	−0.086	0.015	−5.699	1.21 ×10^−8^	***	9.855 ×10^−4^	2.538 ×10^−4^	3.882	1.04 ×10^−4^	***
	25	2nd	0.790	0.151	5.241	1.6 ×10^−7^	***	−0.051	0.005	−9.623	<2 ×10^−16^	***	2.312 ×10^−4^	3.768 ×10^−5^	6.136	8.48 ×10^−10^	***
	30	2nd	0.886	0.149	5.936	2.93 ×10^−9^	***	−0.050	0.005	−9.812	<2 ×10^−16^	***	2.35 ×10^−4^	3.618 ×10^−5^	6.494	8.34 ×10^−11^	***
	35	2nd	0.871	0.147	5.924	3.15 ×10^−9^	***	−0.047	0.005	−9.472	<2 ×10^−16^	***	2.246 ×10^−4^	3.461 ×10^−5^	6.491	8.55 ×10^−11^	***
4th larvae	15	1st	0.471	0.099	4.776	1.790 ×10^−6^	***	−0.013	0.001	−10.120	<2 ×10^−16^	***	1.963 ×10^−5^	3.485 ×10^−6^	5.632	1.78 ×10^−8^	***
	20	1st	1.888	0.105	17.932	<2 ×10^−16^	***	−0.019	0.001	−15.140	<2 ×10^−16^	***	3.131 ×10^−5^	3.339 ×10^−6^	9.377	<2 ×10^−16^	***
	25	1st	3.117	0.131	23.840	<2 ×10^−16^	***	−0.023	0.001	−16.300	<2 ×10^−16^	***	3.704 ×10^−5^	3.635 ×10^−6^	10.190	<2 ×10^−16^	***
	30	1st	5.591	0.393	14.239	<2 ×10^−16^	***	−0.060	0.007	−8.378	<2 ×10^−16^	***	2.769 ×10^−4^	3.949 ×10^−5^	7.012	2.36 ×10^−12^	***
	35	1st	9.873	0.499	19.790	<2 ×10^−16^	***	−0.064	0.004	−14.760	<2 ×10^−16^	***	1.059 ×10^−4^	9.098 ×10^−6^	11.640	<2 ×10^−16^	***
	15	2nd	0.950	0.152	6.271	3.59 ×10^−10^	***	−0.055	0.005	−10.472	<2 ×10^−16^	***	2.619 ×10^−4^	3.766 ×10^−5^	6.955	3.52 ×10^−12^	***
	20	2nd	1.319	0.151	8.753	<2 ×10^−16^	***	−0.053	0.005	−10.653	<2 ×10^−16^	***	2.502 ×10^−4^	3.431 ×10^−5^	7.292	3.06 ×10^−13^	***
	25	2nd	1.865	0.160	11.654	<2 ×10^−16^	***	−0.053	0.005	−10.654	<2 ×10^−16^	***	2.342 ×10^−4^	3.361 ×10^−5^	6.967	3.23 ×10^−12^	***
	30	2nd	2.563	0.183	14.023	<2 ×10^−16^	***	−0.055	0.005	−10.338	<2 ×10^−16^	***	2.263 ×10^−4^	3.449 ×10^−5^	6.563	5.27 ×10^−11^	***
	35	2nd	3.061	0.208	14.688	<2 ×10^−16^	***	−0.055	0.006	−9.608	<2 ×10^−16^	***	2.266 ×10^−4^	3.622 ×10^−5^	6.257	3.94 ×10^−10^	***
	15	3rd	0.805	0.237	3.393	6.91 ×10^−4^	***	−0.110	0.019	−5.786	7.19 ×10^−9^	***	1.2165 ×10^−3^	3.241 ×10^−4^	3.754	1.74 ×10^−4^	***
	20	3rd	1.839	0.259	7.110	1.16 ×10^−12^	***	−0.128	0.019	−6.852	7.27 ×10^−12^	***	1.4197 ×10^−3^	3.026 ×10^−4^	4.692	2.7 ×10^−6^	***
	25	3rd	2.625	0.307	8.552	<2 ×10^−16^	***	−0.128	0.020	−6.354	2.1 ×10^−10^	***	1.2858 ×10^−3^	3.121 ×10^−4^	4.120	3.79 ×10^−5^	***
	30	3rd	2.949	0.347	8.507	<2 ×10^−16^	***	−0.114	0.022	−5.253	1.5 ×10^−7^	***	1.0405 ×10^−3^	3.267 ×10^−4^	3.185	1.45 ×10^−3^	**
	35	3rd	3.079	0.338	9.117	<2 ×10^−16^	***	−0.148	0.021	−6.877	6.12 ×10^−12^	***	1.5525 ×10^−3^	3.256 ×10^−4^	4.769	1.86 ×10^−6^	***
Male	15	1st	0.552	0.178	3.106	1.899 ×10^−3^	***	−0.025	0.004	−6.163	7.13 ×10^−10^	***	1.091 ×10^−4^	2.63 ×10^−5^	4.148	3.36 ×10^−5^	***
	20	1st	1.190	0.099	11.989	<2 ×10^−16^	***	−0.016	0.001	−13.152	<2 ×10^−16^	***	2.617 ×10^−5^	3.364 ×10^−6^	7.778	7.35 ×10^−15^	***
	25	1st	1.818	0.105	17.350	<2 ×10^−16^	***	−0.017	0.001	−14.013	<2 ×10^−16^	***	2.759 ×10^−5^	3.307 ×10^−6^	8.344	<2 ×10^−16^	***
	30	1st	2.657	0.124	21.406	<2 ×10^−16^	***	−0.018	0.001	−12.779	<2 ×10^−16^	***	2.517 ×10^−5^	3.508 ×10^−6^	7.173	7.34 ×10^−13^	***
	35	1st	4.340	0.178	24.414	<2 ×10^−16^	***	−0.027	0.002	−15.069	<2 ×10^−16^	***	4.042 ×10^−5^	4.331 ×10^−6^	9.333	<2 ×10^−16^	***
	15	2nd	0.551	0.154	3.573	3.53 ^×10−4^	***	−0.054	0.006	−9.690	<2 ×10^−16^	***	2.603 ×10^−4^	4.04 ×10^−5^	6.443	1.17 ×10^−10^	***
	20	2nd	0.867	0.148	5.878	4.16 ×10^−9^	***	−0.046	0.005	−9.302	<2 ×10^−16^	***	2.149 ×10^−4^	3.494 ×10^−5^	6.152	7.65 ×10^−10^	***
	25	2nd	1.795	0.159	11.313	<2 ×10^−16^	***	−0.052	0.005	−10.564	<2 ×10^−16^	***	2.298 ×10^−4^	3.376 ×10^−5^	6.808	9.9 ×10^−12^	***
	30	2nd	1.346	0.153	8.830	<2 ×10^−16^	***	−0.057	0.005	−11.199	<2 ×10^−16^	***	2.706 ×10^−4^	3.565 ×10^−5^	7.591	3.18 ×10^−14^	***
	35	2nd	2.667	0.183	14.559	<2 ×10^−16^	***	−0.064	0.005	−11.884	<2 ×10^−16^	***	2.772 ×10^−4^	3.549 ×10^−5^	7.811	5.68 ×10^−15^	***
	15	3rd	0.849	0.375	2.264	2.358 ×10^−2^	*	−0.243	0.060	−4.048	5.16 ×10^−5^	***	7.265 ×10^−3^	2.402 ×10^−3^	3.025	2.49 ×10^−3^	**
	20	3rd	1.828	0.259	7.054	1.73 ×10^−12^	***	−0.143	0.019	−7.394	1.43 ×10^−13^	***	1.5842 ×10^−3^	3.194 ×10^−4^	4.959	7.07 ×10^−7^	***
	25	3rd	1.848	0.260	7.106	1.19 ×10^−12^	***	−0.128	0.019	−6.769	1.3 ×10^−11^	***	1.36 ×10^−3^	3.069 ×10^−4^	4.431	9.37 ×10^−6^	***
	30	3rd	3.376	0.354	9.534	<2 ×10^−16^	***	−0.171	0.023	−7.577	3.53 ×10^−14^	***	1.749 ×10^−3^	3.408 ×10^−4^	5.132	2.87 ×10^−7^	***
	35	3rd	1.653	0.259	6.392	1.64 ×10^−10^	***	−0.076	0.018	−4.231	2.33 ×10^−5^	***	6.251 ×10^−4^	2.871 ×10^−4^	2.177	2.95 ×10^−2^	*
Female	15	1st	1.508	0.102	14.823	<2 ×10^−16^	***	−0.018	0.001	−14.564	<2 ×10^−16^	***	2.932 ×10^−5^	3.4 ×10^−6^	8.624	<2 ×10^−16^	***
	20	1st	2.680	0.118	22.740	<2 ×10^−16^	***	−0.024	0.001	−17.550	<2 ×10^−16^	***	3.981 ×10^−5^	3.494 ×10^−6^	11.390	<2 ×10^−16^	***
	25	1st	4.584	0.180	25.440	<2 ×10^−16^	***	−0.031	0.002	−16.840	<2 ×10^−16^	***	4.738 ×10^−5^	4.373 ×10^−6^	10.840	<2 ×10^−16^	***
	30	1st	6.669	0.308	21.666	<2 ×10^−16^	***	−0.040	0.003	−13.988	<2 ×10^−16^	***	6.12 ×10^−5^	6.316 ×10^−6^	9.689	<2 ×10^−16^	***
	35	1st	17.670	1.131	15.600	<2 ×10^−16^	***	−0.117	0.009	−12.890	<2 ×10^−16^	***	2.011 ×10^−4^	1.794 ×10^−5^	11.210	<2 ×10^−16^	***
	15	2nd	0.599	0.149	4.024	5.72 ×10^−5^	***	−0.045	0.005	−8.698	<2 ×10^−16^	***	2.037 ×10^−4^	3.661 ×10^−5^	5.564	2.64 ×10^−8^	***
	20	2nd	1.054	0.146	7.209	5.63 ×10^−13^	***	−0.037	0.005	−7.980	1.47 ×10^−15^	***	1.589 ×10^−4^	3.236 ×10^−5^	4.911	9.08 ×10^−7^	***
	25	2nd	1.647	0.155	10.605	<2 ×10^−16^	***	−0.045	0.005	−9.319	<2 ×10^−16^	***	1.971 ×10^−4^	3.232 ×10^−5^	6.100	1.06 ×10^−9^	***
	30	2nd	2.449	0.179	13.699	<2 ×10^−16^	***	−0.052	0.005	−10.108	<2 ×10^−16^	***	2.28 ×10^−4^	3.38 ×10^−5^	6.747	1.51 ×10^−11^	***
	35	2nd	3.710	0.241	15.385	<2 ×10^−16^	***	−0.065	0.006	−10.297	<2 ×10^−16^	***	2.816 ×10^−4^	3.923 ×10^−5^	7.178	7.06 ×10^−13^	***
	15	3rd	0.629	0.233	2.702	6.9 ×10^−3^	**	−0.086	0.018	−4.714	2.43 ×10^−6^	***	8.777 ×10^−4^	3.081 ×10^−4^	2.849	4.39 ×10^−3^	**
	20	3rd	2.046	0.268	7.621	2.51 ×10^−14^	***	−0.127	0.019	−6.740	1.58 ×10^−11^	***	1.3819 ×10^−3^	3.016 ×10^−4^	4.582	4.6 ×10^−6^	***
	25	3rd	2.606	0.309	8.422	<2 ×10^−16^	***	−0.119	0.020	−5.928	3.06 ×10^−9^	***	1.2178 ×10^−3^	3.099 ×10^−4^	3.930	8.51 ×10^−5^	***
	30	3rd	3.298	0.371	8.892	<2 ×10^−16^	***	−0.135	0.023	−5.938	2.89 ×10^−9^	***	1.332 ×10^−3^	3.386 ×10^−4^	3.934	8.37 ×10^−5^	***
	35	3rd	4.437	0.479	9.267	<2 ×10^−16^	***	−0.187	0.028	−6.769	1.3 ×10^−11^	***	1.8606 ×10^−3^	3.918 ×10^−4^	4.749	2.05 ×10^−6^	***

Not all predator stages could successfully utilize S. litura as their prey. There was no predation on 2nd and 3rd instars of prey S. litura by 1st, 2nd, and 3rd instars of H. axyridis, respectively. The densities offered of each prey size were different. The mean weights of 1st, 2nd, and 3rd instars of prey S. litura were 7.31 ×10^−05^ ± 2.99 ×10^−05^, 2.95 ×10^−04^ ± 1.20 ×10^−04^, and 1.37 ×10^−03^ ± 5.58 ×10^−04^ g per larvae, respectively. *, **, and *** represent significance at 0.05, 0.01, and 0.001 probability levels. Pr(>|z|) column represents the p-value associated with the value in the z-value column. If the p-value is less than a certain significance level (e.g., α = 0.05), then this indicates that the predictor variable has a statistically significant relationship with the response variable in the model.

*Number of replications (N) = 10.*

*The ^a^ indicates the level of significance at P <0.05*.

Finally, we calculated the maximum predation rate from the estimates of *T*_*h*_. The ratio of 1/*T*_*h*_ represents the maximum predation rate considering a predator that spends all its time handling (i.e., 24 h here). The CIs for maximum predation rate were obtained from a set of 20 simulated samples, and 50 datasets (i.e., 50 simulated replicates) were generated for this study by using “simaR” R software package (Benhadi-Marín et al., 2018). Again, significant differences in maximum predation rates among the temperatures were calculated by non-overlapping CIs.

## Results

### Predation of *H. axyridis* on *S. litura*

**Figure 1:**
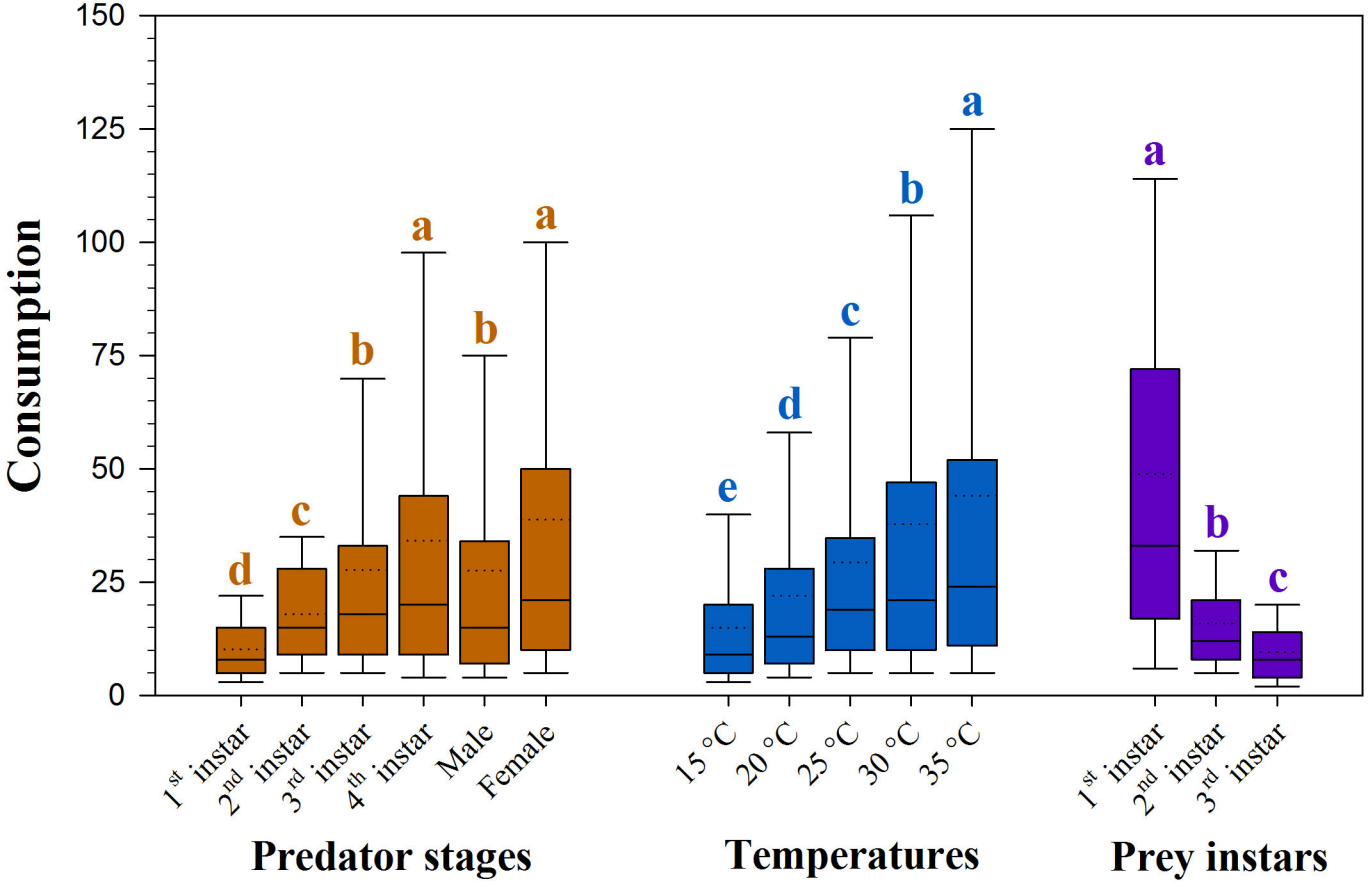
Consumption of prey *Spodoptera litura* by *Harmonia axyridis* at five temperatures and three prey instars. Box plots show the range of data (lower and upper quartiles), median (line), and mean (dotted line), and different lowercase letters above the whisker caps indicate significant differences among group mean values according to 95% CI. Number of replications (N) = 10.

Predation of *H. axyridis* on the larvae of *S. litura* was dependent upon temperature and the size of both predator and prey (all *P* <0.05, Supplementary Table 1). The rate of predation increased with warming and with advances in the predator stage; however, predation decreased with advances in the prey stage (Figure 1).

Effects of temperature, predator stage, prey density, and their interactions were significant on the prey instars tested (*P* <0.05, Supplementary Table 2), except for predator stage and prey density interaction on 3rd prey instars (*P* = 0.1156).

**Figure 2:**
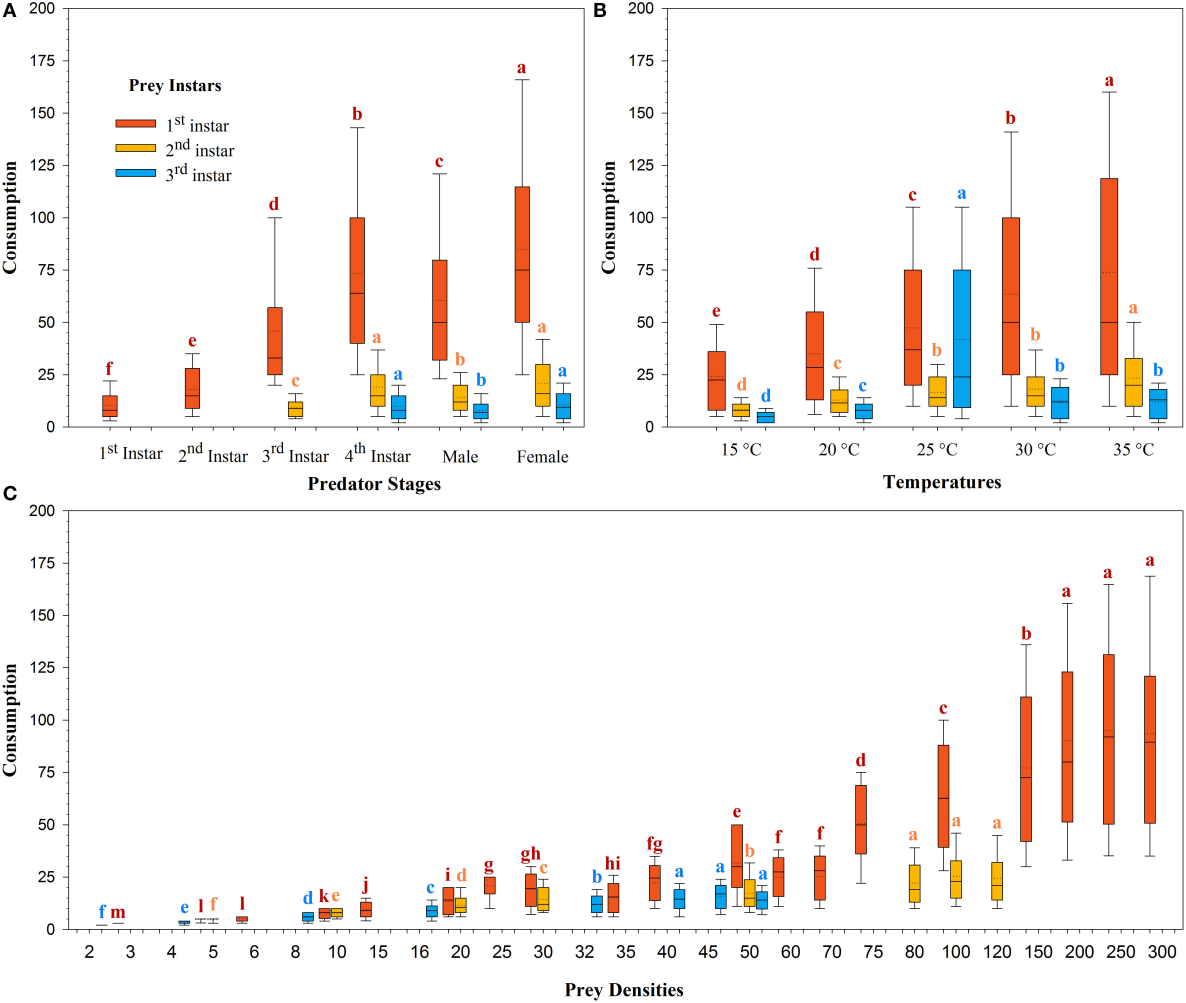
Consumption of prey *Spodoptera litura* by *Harmonia axyridis* at five temperatures, according to predator stage **(A)**, temperatures **(B)**, and prey densities offered **(C)**. Box plots show the range of data (lower and upper quartiles), median (line), and mean (dotted line), and different lowercase letters above the whisker caps indicate significant differences among group mean values according to 95% CI. Number of replications (N) = 10.

The magnitude of predation was different for the same predator in the three prey systems across the temperatures tested (Figure 2). The mean predation rate for females decreased from 84.88 ± 2.47 larvae/arena to 10.83 ± 0.36 larvae/arena with advances in the prey size from the 1st to the 3rd instar (Figure 2A). The mean rate of prey predation increased from 35.05 ± 1.16 larvae/arena to 73.92 ± 2.58 larvae/arena on the 1st instar of prey *S. litura*, 8.50 ± 0.23 larvae/arena to 23.43 ± 0.88 larvae/arena on the 2nd instar of prey of *S. litura*, and 4.86 ± 0.17 larvae/arena to 12.53 ± 0.49 larvae/arena on the 3rd instar of prey *S. litura* as the temperature increased from 15 to 35°C. Predation was lowest at 15 and 20°C, intermediate at 25 and 30°C, and greatest at 35°C on the 1st and 2nd instars of prey *S. litura*, whereas on the 3rd instar of prey *S. litura*, predation was found to be the lowest at 15 and 20°C, intermediate at 30°C and 35°C, and greatest at 25°C (Figure 2B).

The rate of prey predation was much higher when feeding on the 1st instar of *S. litura* than on the 2nd and 3rd instars of *S. litura* (Figure 1). The highest predation was noted for females, followed by 4th instar and adult males against 1st instar of *S. litura*. The predation rate of the 4th instar and female *H. axyridis* was similar in the 2nd and 3rd instars of *S. litura*. Initial instars of *H. axyridis* (i.e., 1st and 2nd instar) showed the lowest predation against the 1st instar of *S. litura*. Likewise, 3rd instar of *H. axyridis* exhibited the lowest predation rate against the 2nd instar of *S. litura* (Figure 2A).

The predation rate increased with the initial density of prey offered. The maximum predation rate was noted at densities of 250 (i.e., 94.93 ± 3.37), 300 (i.e., 93.55 ± 3.42), and 200 (i.e., 90.12 ± 3.20) larvae/arena against 1st instar of *S. litura*. On the 2nd instar of *S. litura*, the predation was greatest at a density of 100 (i.e., 25.41 ± 0.93), 120 (i.e., 24.47 ± 0.94), and 80 (i.e., 22.12 ± 0.83) larvae/arena, whereas on 3rd instar of *S. litura*, the predation was greatest at a density of 45 (i.e., 15.84 ± 0.50), 40 (i.e., 14.27 ± 0.46), and 50 (i.e., 14.00 ± 0.44) (Figure 2C).

### Functional Response

**Figure 3:**
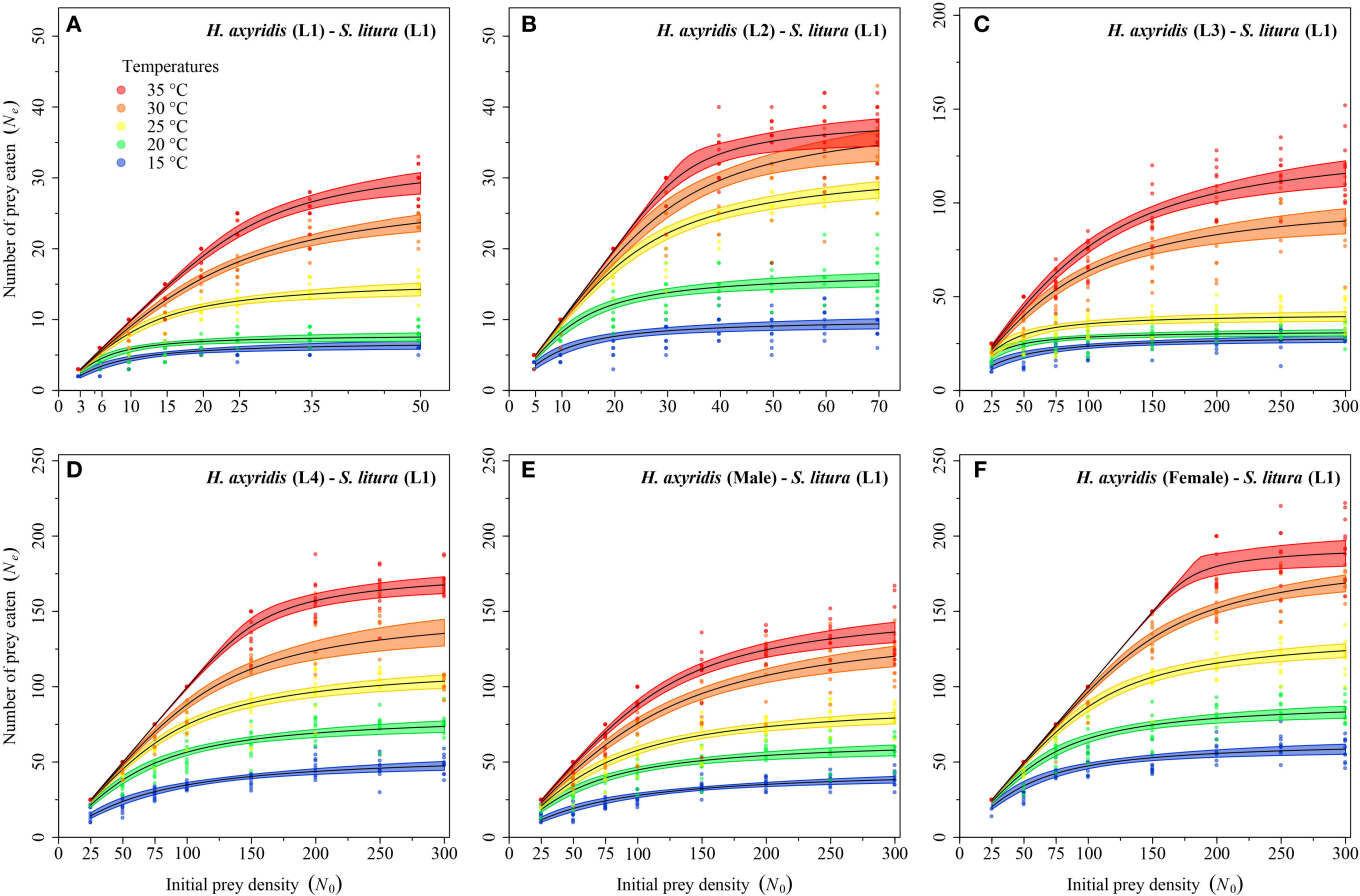
Functional response of different stages of *Harmonia axyridis* [L1-Female **(A–F)**] foraging on 1st instar of *Spodoptera litura*. Dots represent the observed numbers of prey consumed at each initial prey density, and black lines were predicted by the Roger's random predator equation, while the shaded areas represent the limits of the 95% confidence intervals. Number of replications (N) = 10.

**Figure 4:**
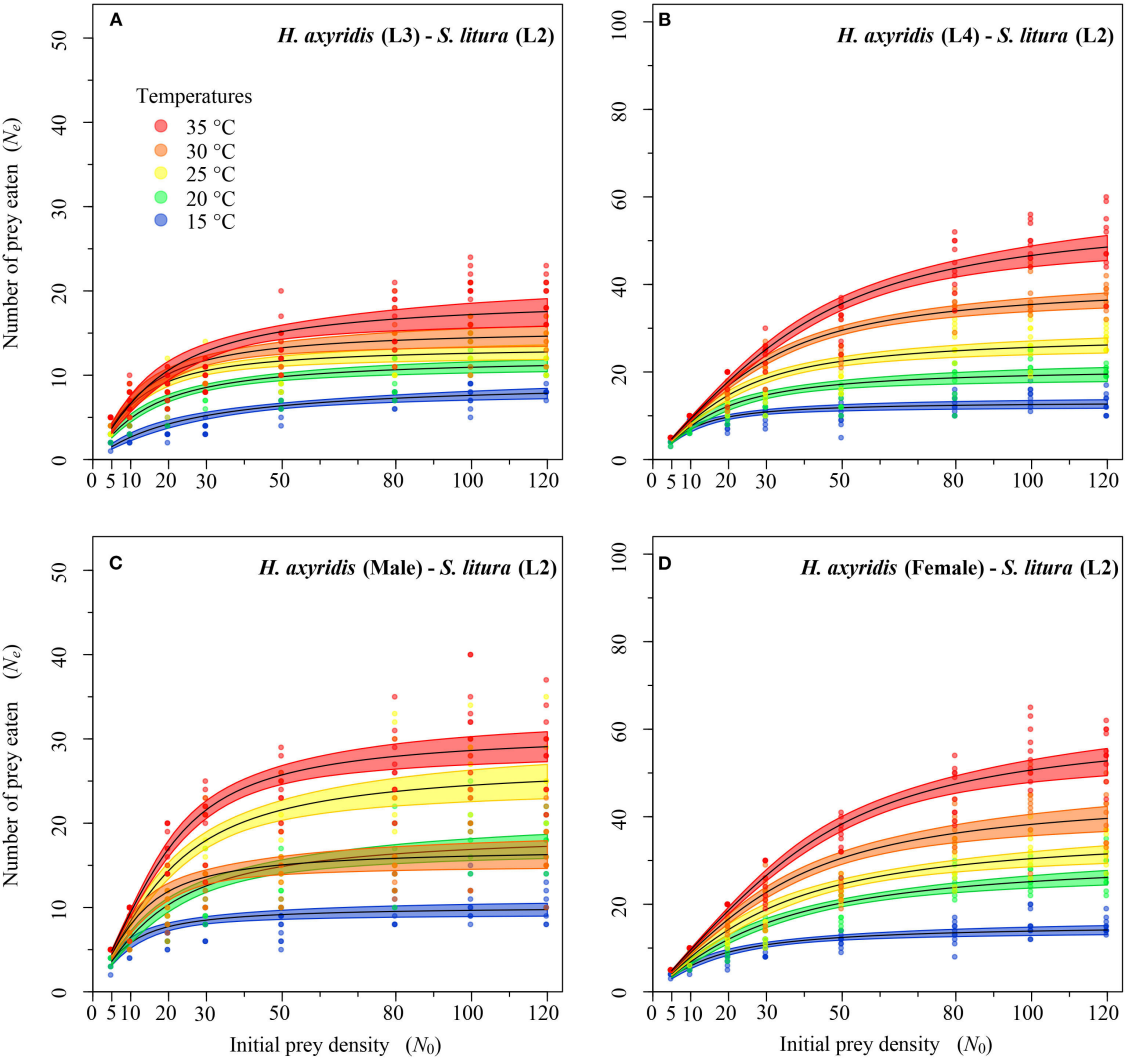
Functional response of different stages of *Harmonia axyridis* [L3-Female (**A–D**)] foraging on 2nd instar of *Spodoptera litura*. Dots represent the observed numbers of prey consumed at each initial prey density, and black lines were predicted by the Roger's random predator equation, while the shaded areas represent the limits of the 95% confidence intervals. Number of replications (N) = 10.

**Figure 5:**
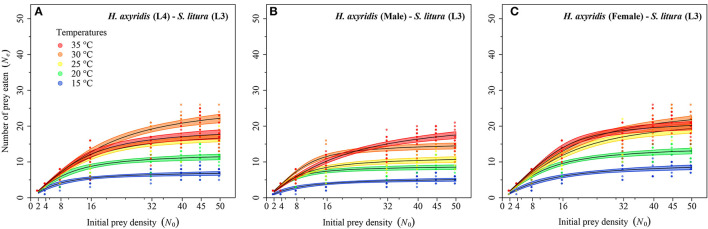
Functional response of different stages of *Harmonia axyridis* [L4, Female **(A–C)**] foraging on 3rd instar *Spodoptera litura*. Dots represent the observed numbers of prey consumed at each initial prey density, and black lines were predicted by the Roger's random predator equation, while the shaded areas represent the limits of the 95% confidence intervals. Number of replications (N) = 10.

Logistic regression showed that all predator stages exhibited type II functional responses at all temperatures. This is because the linear estimates of Equation 1 were significantly negative for all stages of predator except for 1st instar and adult female at 35°C (Table 1). The curves show that initially at low prey densities, predation quickly increased across all predatory stages at all tested temperatures but leveled off with a further increase in prey density (Figures 3–5). The proportion of all stages of *S. litura* consumed by *H. axyridis* decreased monotonically with prey density offered (Supplementary Figures 1A–M).

**Figure 6:**
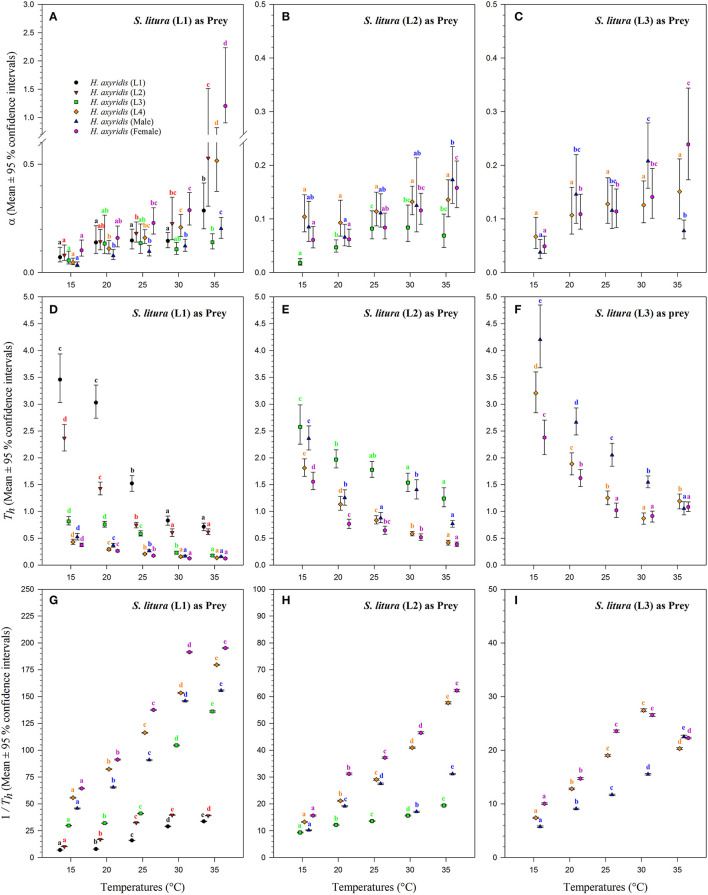
Functional response coefficients of *Harmonia axyridis* foraging on 1st, 2nd, and 3rd instars of *Spodoptera litura* under different temperatures. Mean ± 95% confidence intervals of space clearance rate [*a* **(A–C)**], handling time [*T*_h_ **(D–F)**], and maximum predation rate [1/*T*_h_ **(G–I)**] were estimated from bootstrapped data (*n* = 10,000). Different lowercase letters above the caps indicate significant differences in *H. axyridis* stages among the temperatures according to 95% CI. Number of replications (N) = 10.

The magnitude of the functional response of *H. axyridis* was considerably different between temperatures and prey instars offered (Figure 6). The space clearance rate decreased (Figures 6A–C) and handling time increased (Figures 6D–F) as the prey size increased. For 1st instar of *S. litura*, the highest space clearance rate (α) and lowest handling time (*T*_*h*_) was found for females at 35°C. The lowest space clearance rate was shown by the 3rd instar of predator (i.e., 0.018 ± 0.003 h^−1^) at 15°C against the 2nd instar of prey larvae. The highest handling time against the 1st instar of *S. litura* was exhibited by the 1st instar of *H. axyridis* (i.e., 3.457 ± 0.309 h) at 15°C. The highest space clearance rate (i.e., 0.173 ± 0.017 h^−1^) and lowest handling time (i.e., 0.386 ± 0.013 h) against the 2nd instar of prey *S. litura* were noted for males and females, respectively, at 35°C. The highest space clearance rate (i.e., 0.239 ± 0.035 h^−1^) and the lowest handling time (i.e., 0.871 ± 0.047 h) on the 3rd instar of *S. litura* were exhibited by female and 4th instar of *H. axyridis* at 35 and 30°C, respectively. The handling time of the 4th instar of *H. axyridis* was lower than that of the female at the 3rd instar of prey at 30°C.

The space clearance rate of 4th instar larvae was higher on the 1st instar of prey (i.e., 0.517 ± 0.039 h^−1^) than on the 2nd (i.e., 0.136 ± 0.009 h^−1^) and 3rd instars of *S. litura* (i.e., 0.150 ± 0.02 h^−1^) at 35°C. Similarly, the handling time of 4th instar larvae was lower on the 1st instar (i.e., 0.134 ± 0.002 h) than on the 2nd (i.e., 0.416 ± 0.015 h) and 3rd instar larvae of *S. litura* (i.e., 1.192 ± 0.060 h) at 35°C. At 15°C, the space clearance rates of the 4th instar and adult males were higher than that of the 2nd instar and 1st instar of *S. litura*.

The maximum predation rate (1/*T*_h_) increased with warming and *H. axyridis* age but decreased with prey age (Figures 6G–I). Overall, the maximum rate was noted for females, followed by 4th instar and adult males against all stages of the prey. For the 1st instar of prey *S. litura*, the maximum predation rate of females increased from 64.370 ± 0.184 to 195.209 ± 0.238 as the temperature increased from 15 to 35°C. As the prey size increased from the 2nd to the 3rd instar, the maximum predation rate of females and males decreased. The maximum predation rate for both 4th instars also was dependent on the prey size-, and it decreased as the prey size increased. For the 1st instar of prey *S. litura*, the maximum predation rates were calculated for the 4th instar, males, and females (i.e., 179.535 ± 0.255, 155.651 ± 0.272, and 195.209 ± 0.238, respectively), which were also greater at 35°C.

## Discussion

The quantification of biotic interaction strengths is fundamental for understanding population processes (Shaver et al., 2000) and the trophic interactions structuring the food webs (Anderson et al., 2001). *H. axyridis* is among a group of predators with a worldwide distribution and depends on trophic interactions with agricultural pests. This study demonstrated the broad dependence of the foraging rates of *H. axyridis* on temperature, prey densities, and both prey and predator body masses (stages). *H. axyridis* in all growth stages readily preyed upon 1st instar of prey *S. litura*, but 1st, 2nd, and 3rd instar predators were unable to consume the larger 2nd and 3rd instars of prey, suggesting that predators at initial stages lack the ability to capture or handle bigger prey that is generally more aggressive over their smaller counterparts. Adults (male and female) and the 4th instar of *H. axyridis* could feed upon all prey sizes, but predation substantially declined with increasing prey size. The 4th instar and adult female *H. axyridis* consumed more than their male counterparts. Both larval and adult predators foraged more with increasing temperature. This may be explained by the acceleration of biological rates (i.e., metabolism) under warm conditions, which increases both the capacity to do biological work and the demand for resources. Our results are consistent with previous reports (Islam et al., 2020, 2021) that showed heightened predation with warming by the 4th instar and female *H. axyridis* against *S. litura* eggs and *Acyrthosiphon pisum* (Hemiptera: Aphididae) (Harris) and also that they foraged relatively more than males and younger predators. Similar results were shown for *Harmonia dimidiata* (F.) (Coleoptera: Coccinellidae) preying on *Aphis gossypii* Glover (Hemiptera: Aphididae) (Yu et al., 2013) and for *Podisus maculiventris* (Say) and *Podisus nigrispinus* (Dallas) (Heteroptera: Pentatomidae) preying upon immature *Spodoptera exigua* (Hubner) (Lepidoptera: Noctuidae) (Mohaghegh et al., 2001).

Our results indicate that the functional response of *H. axyridis* on *S. litura* larvae was type II across prey and predator body size (stages) and temperature. Our results are in line with some previous studies with coccinellids where warming did not change the type of functional response, including *Scymnus levaillanti* Mulsant, *Adalia bipunctata* L., and *Cycloneda sanguinea* (L.) (Coleoptera: Coccinellidae) feeding on *Aphis gossypii* Glover and *Myzus persicae* (Sulzer) (Hemiptera: Aphididae) (Işikber, 2005; Jalali et al., 2010). However, our results are different than other reports, where *Eupeodes corollae* (Fabricius) (Diptera: Syrphidae) preying on *Spodoptera frugiperda* (J. E. Smith) (Lepidoptera: Noctuidae) larvae (Li et al., 2021) showed a type III functional response (density-dependent predation rate) to initial instars of *S. frugiperda* on fresh maize leaves. The authors suggested the proportion of prey consumed by predators accelerated initially owing to better prey searching by predators in low-density patches. Other mechanisms generating type III functional responses include predator learning, prey switching, predator–prey encounter rates and movements, prey detectability, and intraguild predation (Verdy and Amarasekare, 2010; DeLong, 2021; Bruzzone et al., 2022). These mechanisms, however, do not seem to operate in our predator–prey system, suggesting the need to test for individual predator–prey systems for a better understanding of how temperature drives food webs.

The parameters space clearance rate and handling time determine the height and shape of functional responses (Pervez, 2005; DeLong, 2021). The space clearance rate describes the ability of a predator to capture prey and determines how steeply the functional response curve rises with the increasing prey density. The handling time includes all-time costs that interrupt further searching, including evaluating, catching, pursuit, and predation (Pervez, 2005; DeLong, 2021). Thus, the response of these two parameters to temperature is critical for understanding predator–prey interactions in different locations and in response to climate change. Our findings demonstrate that *H. axyridis* predators have increased space clearance rates at higher temperatures, indicating that low-density prey populations in warmer environments can be reduced more successfully. Further increases in temperature, however, might be expected to reduce space clearance rate, since predators of many species have their highest feeding rates at intermediate temperatures (Englund et al., 2011; Uszko et al., 2017; Uiterwaal and DeLong, 2020). Typically, predators spend more time handling prey at lower temperatures than at higher temperatures (Mohaghegh et al., 2001; DeLong and Uiterwaal, 2021), and this is consistent with our findings. In our findings, linear estimates of 1st instar and female *H. axyridis* also exhibited a type II response at 35°C, although the parameters were not significant. The temperature was found to impact changes in the linear estimates, driving higher magnitude functional response by decreasing handling times. Our results are consistent with previous findings where 4th instar and female *H. axyridis* showed a type II functional response against *A. pisum* at 30°C even though the linear estimates were not significant (Islam et al., 2021). This temperature effect suggests increased predator effectiveness at warmer temperatures at high prey densities as well. Overall, warming appears to likely improve the effectiveness of *H. axyridis* on *S. litura*, but the temperature at which foraging rates begin to decline is currently not known.

Variation in functional response parameters across prey stages suggests that *H. axyridis* will be effective at limiting only a portion of the *S. litura* population. Specifically, the space clearance rate of *H. axyridis* decreased with the larval sizes of *S. litura*, and handling time increased as prey size increased from the 1st to 3rd instar, indicating that *H. axyridis* is most effective at limiting smaller and younger *S. litura* individuals. It is likely more difficult and time consuming for the predator to capture large size prey, possibly due to physical feeding limitations (i.e., gape limitation), while predators may be unwilling to consume prey that is too small due to their poor nutritional value. A possible explanation is that larger prey (2nd and 3rd instars) are more aggressive and difficult to capture. Although observations of the predator's feeding behavior in this study showed that prey of all sizes was swallowed completely, in some cases, 4th instar and adult *H. axyridis* regurgitated large *S. litura* larvae several times before finally ingesting them. This behavior could explain the shorter handling times for small prey (Kreuzinger-Janik et al., 2019). The success of any natural enemy, however, cannot be traced solely to its functional response to the target insect in laboratory settings. Natural enemy behavior and efficiency can be affected by the limited size of the arena used in laboratory experiments compared to field circumstances, and also with respect to spatial complexity in nature, host features, biotic and abiotic influences, and numerical response. All these variables must be explored for the species under study for further development of successful biocontrol programs.

In conclusion, the present study found that the functional response of *H. axyridis* foraging on *S. litura* larvae was temperature-dependent and type II at all temperatures and stages of the prey. Variation in functional responses suggests that the most effective use of these predators is early in the season to cope with early pest infestation. In the tropics where the infestations of S. *litura* start in the hot season, this predator may provide effective control due to the steeper and higher functional response in warmer temperatures. Where prey size is large and thus difficult to control for *H. axyridis*, the use of botanicals that can physiologically stress the pest and possibly reduce their defense could be a viable method for increasing susceptibility to predators. More generally, this strong temperature and body size influence on functional response parameters can lead to important changes in predator–prey relationship, population dynamics, and food web interactions (Sentis et al., 2012). However, the nature of these ecological interactions can be very complex and difficult to predict from laboratory studies. It should be expected that warmer temperatures may favor the development of both the prey and predator but with impacts and concerns that should vary widely. *S. litura* can develop at a much faster rate under warmer temperatures, but aphids have a weaker developmental response to temperature (Fand et al., 2015; Islam et al., 2021). The development of *H. axyridis* may be fostered with warmer temperatures, but the life cycle may be disturbed, as the temperatures beyond 35°C did not allow the hatching of *H. axyridis* eggs (preliminary experiment results). Furthermore, increased development with warmer temperatures reduces the time of the developmental stages, resulting in the predator consuming more prey during a single unit of time compared to its entire life stage. As global warming represents a global challenge, and as parts of the tropics and Asia often experience temperatures above 35°C during summer months (Ma et al., 2021), the release of this predator when temperatures have gone beyond 35°C may affect biocontrol sustainability through effects on predator development and biology. We therefore suggest that current findings should be followed with great caution for sustained control of *S. litura*, particularly in protected crops and plantations, and when temperatures are between 25 and 35°C. Further studies should address the development and biology of *H. axyridis* foraging on the eggs and larvae of prey *S. litura* for developing a more effective biocontrol strategy to manage this serious pest.

## Data Availability Statement

The original contributions presented in the study are included in the article/Supplementary Material, further inquiries can be directed to the corresponding author/s.

## Author Contributions

FS conceived the idea. XZ supervised the laboratory trials. FS and XZ provided the necessary guidance to conduct this experiment. YI performed the experiments, analyzed the functional response data, and wrote the manuscript partially. FS and AG analyzed the predation and functional response data, prepared figure visuals, and wrote the final manuscript. JD reviewed and edited the manuscript. All authors critically reviewed, discussed, and approved the manuscript for publication.

## Funding

This research was funded by the National Key R and D Program of China (2017YFD0201000), the National Natural Science Foundation of China (Grant No. 31872023), and the Key Research Program of Hubei Tobacco Company (027Y2018-008).

## Conflict of Interest

The authors declare that the research was conducted in the absence of any commercial or financial relationships that could be construed as a potential conflict of interest.

## Publisher's Note

All claims expressed in this article are solely those of the authors and do not necessarily represent those of their affiliated organizations, or those of the publisher, the editors and the reviewers. Any product that may be evaluated in this article, or claim that may be made by its manufacturer, is not guaranteed or endorsed by the publisher.
