# Supplementary material for: Functional Response of Harmonia axyridis to the Larvae of Spodoptera litura: The Combined Effect of Temperatures and Prey Instars
Source: Front Plant Sci. 2022 Jul 1;13:849574. doi: 10.3389/fpls.2022.849574 (PMC9284266; doi:10.3389/fpls.2022.849574)
Supplement: Supplementary file 1 [file Data_Sheet_1.docx]

Supplementary material for:

**Functional response of *Harmonia axyridis* on the larvae of *Spodoptera litura*: The combined effect of temperatures and prey instars**

Yasir Islam^1^, Farhan Mahmood Shah ^2,a*^, Ali Güncan^3,^ John Paul DeLong^4^ and Xingmiao Zhou^1,^*

^1^Hubei Insect Resources Utilization and Sustainable Pest Management Key Laboratory, College of Plant Science and Technology, Huazhong Agricultural University, Wuhan 430070, China

^2^ Department of Entomology, Faculty of Agricultural Sciences and Technology, Bahauddin Zakariya University, Multan 60000, Pakistan (ORCID: 0000-0002-6123-1860)

^3^Department of Plant Protection, Faculty of Agriculture, Ordu University, 52200 Ordu, Turkey (ORCID: 0000-0003-1765-648X, e-mail: guncan.ali@gmail.com)

^4^School of Biological Sciences, University of Nebraska–Lincoln, 68588, USA

^a^Present Address: National Center for Natural Products Research, University of Mississippi, University, MS, USA (ORCID: 0000-0002-6123-1860)

*Correspondence to: FMS: ([farhanshah0009@yahoo.com](mailto:farhanshah0009@yahoo.com); fshah@olemiss.edu); XZ: x[mzhou@mail.hzau.edu.cn](mailto:mzhou@mail.hzau.edu.cn)

**Supplementary Table 1** GLM model results for consumption by larvae and adult *Harmonia axyridis* of prey *Spodoptera litura* at five temperatures and three prey sizes

| Sources | LR *χ^2^* | *df* | *P* |
| --- | --- | --- | --- |
| *Fixed Factors* |  |  |  |
| Predator stage (*P*) | 14670 | 5 | <0.001 |
| Prey stage (*S*) | 3001 | 2 | <0.001 |
| Temperature (*T*) | 37615 | 4 | <0.001 |
| *P* × *S* | 140 | 5 | <0.001 |
| *P* × *T* | 702 | 20 | <0.001 |
| *S* × *T* | 1160 | 8 | <0.001 |
| *P* × *S* × *T* | 492 | 20 | <0.001 |
| Covariate |  |  |  |
| Prey weight | 11324 | 1 | <0.001 |

Not all predator stages could successfully utilize *S. litura* as their prey. There was no predation on 2^nd^ and 3^rd^ instar prey *S. litura* by 1^st^, 2^nd^ and 3^rd^ instar *H. axyridis*, respectively. The densities offered of each prey size were different. The model assumed predator and temperature as the fixed effects and the prey size measured in weights as the covariate structure. The mean weights of 1^st^, 2^nd^ and 3^rd^ instar prey *S. litura* were 7.31×10^-5^ ± 2.99×10^-5^, 2.95×10^-4^ ± 1.20×10^-4^, and 1.37×10^-3^ ± 5.58×10^-4^ g per larvae. Number of replications (N) = 10.

**Supplementary Table 2** GLM model results for consumption by larvae and adult *Harmonia axyridis* of prey *Spodoptera litura* at various temperatures and prey densities within each prey size offered.

|  | Prey 1^st^ | | | Prey 2^nd^ | | | Prey 3^rd^ | | |
| --- | --- | --- | --- | --- | --- | --- | --- | --- | --- |
| Sources | LR *χ^2^* | *df* | *P* | LR *χ^2^* | *df* | *P* | LR *χ^2^* | *df* | *P* |
| ***Fixed Factors*** |  |  |  |  |  |  |  |  |  |
| Predator stage (*P*) | 15443 | 5 | <0.001 | 3382.4 | 3 | <0.001 | 382.14 | 2 | <0.001 |
| Prey density (*D*) | 26946 | 18 | <0.001 | 7868.9 | 7 | <0.001 | 2643.10 | 7 | <0.001 |
| Temperature (*T*) | 36160 | 4 | <0.001 | 4318.7 | 4 | <0.001 | 1923.43 | 4 | <0.001 |
| *P × T* | 1146 | 20 | <0.001 | 512.3 | 12 | <0.001 | 44.29 | 8 | <0.001 |
| *P × D* | 1175 | 24 | <0.001 | 163.1 | 21 | <0.001 | 20.48 | 14 | 0.1156 |
| *T × D* | 2223 | 72 | <0.001 | 226.0 | 28 | <0.001 | 163.45 | 28 | <0.001 |
| *P × T × D* | 1302 | 96 | <0.001 | 190.1 | 84 | <0.001 | 104.02 | 56 | 0.0001 |

The effects were calculated within each prey size offered to the predator. Number of replications (N) = 10.


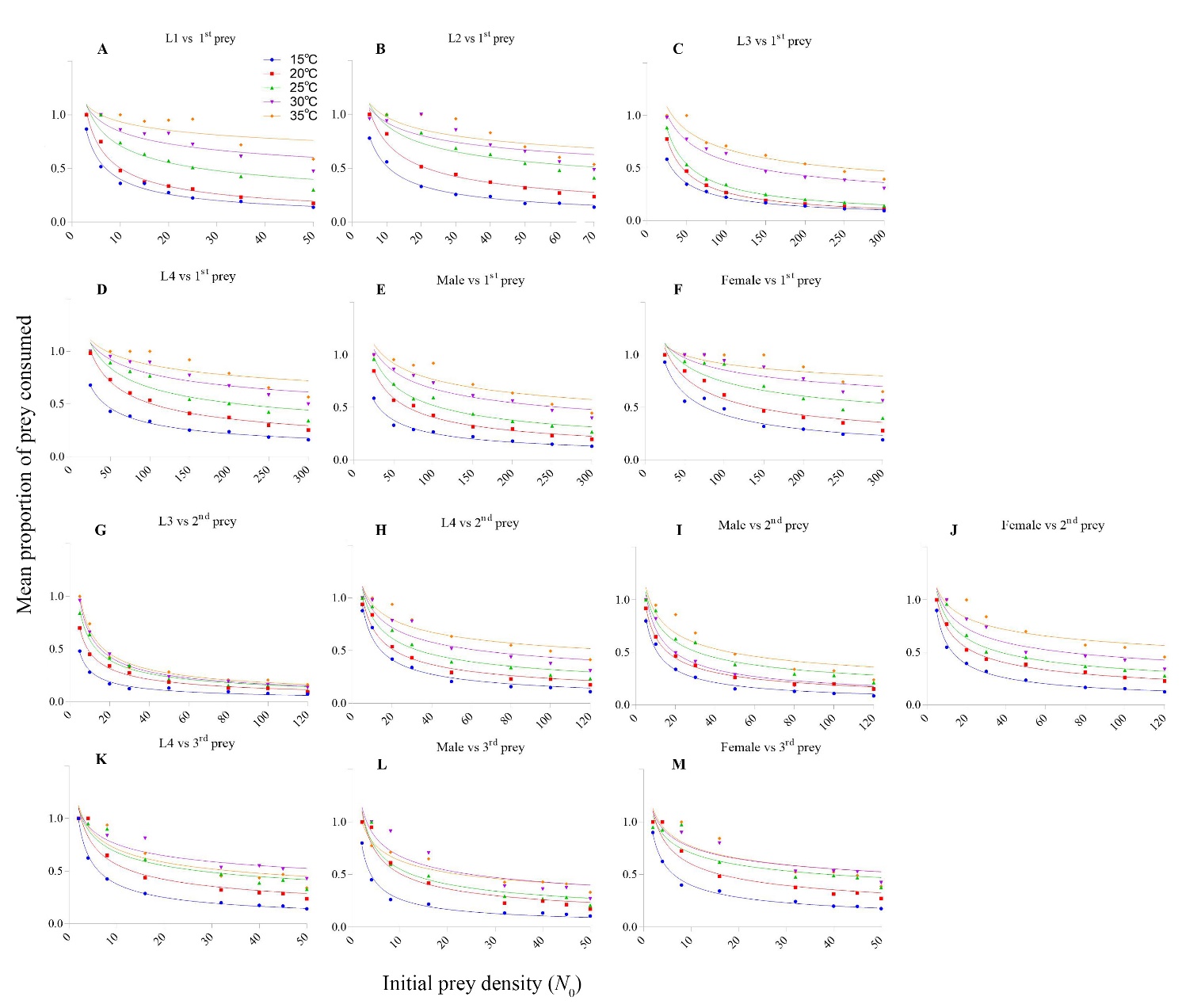


Supplementary Figure 1 Proportion consumed of prey *Spodoptera litura* (1^st^, 2^nd^ and 3^rd^ instar) at various growth stages by *Harmonia axyridis* across five temperatures. Number of replications (N) = 10.
